# Soil habitat condition shapes *Tamarix chinensis* community diversity in the coastal saline-alkali soils

**DOI:** 10.3389/fpls.2023.1156297

**Published:** 2023-04-26

**Authors:** Qianjun Yu, Lizhu Suo, Ji Qi, Yi Wang, Qiuli Hu, Yan Shan, Ying Zhao

**Affiliations:** ^1^ College of Resources and Environmental Engineering, Ludong University, Yantai, China; ^2^ Sericulture Research Institute, Shandong Academy of Agricultural Sciences, Yantai, China

**Keywords:** halophyte, soil salinity, species diversity, coastal wetland, Yellow River Delta (YRD)

## Abstract

**Introduction:**

Unfavorable coastal saline-alkali soil habitats degrade plant community diversity and reduce terrestrial ecological functions. Previous studies have been conducted on the mechanisms by which certain saline-alkali soil properties determine plant community diversity, however, how those properties synergistically affect plant community diversity remains unclear.

**Methods:**

Here, 36 plots of typical *Tamarix chinensis* communities were investigated for a range of parameters at three different distances (10, 20, and 40 km) from the coastline in the Yellow River Delta between 2020 and 2022, and corresponding soil samples were taken and analyzed.

**Results and discussion:**

Our results suggest that although *T. chinensis* density, ground diameter, and canopy coverage significantly increased (*P*<0.05) with increasing distance from the coast, the communities with the most plant species were found at 10 to 20 km distance from the coastline, indicating the effects of soil habitat on *T. chinensis* community diversity. Simpson dominance (species dominance), Margalef (species richness), and Pielou indices (species evenness) differed significantly among the three distances (*P*<0.05) and were significantly correlated with soil sand content, mean soil moisture, and electrical conductivity (*P*<0.05), indicating that soil texture, water, and salinity were the main factors governing *T. chinensis* community diversity. Principal component analysis (PCA) was performed to construct an integrated soil habitat index (SHI) representing the synthesis of the soil texture-water-salinity condition. The estimated SHI quantified a 64.2% variation in the synthetic soil texture-water-salinity condition and was significantly higher at the 10 km distance than at the 40 and 20 km distances. The SHI linearly predicted *T. chinensis* community diversity (R^2^ = 0.12–0.17, *P*<0.05), suggesting that greater SHI (coarser soil texture, wetter soil moisture regime, and higher soil salinity) was found closer to the coast and coincided with higher species dominance and evenness and lower species richness in the *T. chinensis* community. These findings on the relationship between *T. chinensis* communities and soil habitat conditions will be valuable in planning the restoration and protection of the ecological functions of *T. chinensis* shrubs in the Yellow River Delta.

## 1 Introduction

Terrestrial plant community diversity strongly affects ecological functions, i.e., ecosystem productivity, biological elements cycling, and soil carbon sequestration (Miri and Robin, 2021; Shi et al., 2022; Qian et al., 2022; Tao et al., 2022b). In the Yellow River Delta, China’s largest delta, wetland halophyte plants, especially *T. chinensis*, have developed widespread community patches and have made significant contributions to ecological functions due to their high salinity and waterlogging tolerance. Previous studies have reported that soil water, salinity, and nutrients significantly affect the *T. chinensis* plant community in the Yellow River Delta, but they only examined the influence of plant community diversity using a single soil index (Wu et al., 2020; Yang et al., 2021; Zhang et al., 2023). Therefore, they were unable to fully reflect the impact of the soil environment on *T. chinensis* plant community diversity. Given the unique water-salt interactions under seawater intrusion and the young alluvial sediments in the Yellow River Delta (Fu et al., 2021), the soil habitat conditions in which the synthesis of soil texture, water, salinity, and nutrients occurs exhibit great spatial heterogeneity potentially affecting the *T. chinensis* community diversity. However, how and to what extent the integrated soil habitat conditions determine *T. chinensis* community diversity is unclear. New information on the regulating mechanisms of soil habitat conditions will be of considerable value for restoring and protecting plant communities at an operational level in the Yellow River Delta.

Soil habitats in coastal areas are vulnerable to the influence of oceans, rivers, and other external forces. In general, at shorter distances from the coastline, where the soil is highly erodible, the groundwater table is shallow, soil water moisture and salinity are high, and soil nutrients are limited (Young et al., 2011; Osland et al., 2016). Soil texture affects the efficiency of water and nutrient uptake by different plant species, thus shaping the internal composition and diversity of *T. chinensis* communities (Jalota et al., 2010; Renne Rachel et al., 2019; Tran Cuc et al., 2021). Soil moisture affects plant photosynthesis and dry matter accumulation, thus influencing *T. chinensis* community distribution (Christine et al., 2019; Cai et al., 2021; Pinke et al., 2022). High soil salinity inhibits the growth of plants with low salinity tolerance and decreases the diversity of the *T. chinensis* community (Qu et al., 2017). Soil nutrients promote plant growth and development and determine the species composition of *T. chinensis* communities, whereas appropriate soil nutrient content increases plant diversity (Han et al., 2022). Numerous studies have examined the independent effects of soil texture, water content, salinity, and nutrients on plant community diversity. However, how soil habitat conditions change at different distances from the coast and how soil habitat conditions affect soil texture, water, salinity, and nutrients to determine the nature of *T. chinensis* communities remains unclear.

Plant community species diversity is a measure of the complexity of plant community structure and function (Hooper et al., 2005), which reflects the degree of plant community affected by the environment. Li et al. (2018) found that when soil texture gradually changed from clay to sandy loam, plant community diversity tended to decrease; in particular, the Shannon–Wiener index showed a downward trend, but the Simpson dominance index increased in the Taohe Wetland, China (Li et al., 2018). Zhao et al. (2021) found that the Margalef index was positively correlated with increasing soil water content during wetland restoration in Zhangye National Wetland Park, China (Zhao et al., 2021). Gaberscik et al. (2018) found that plant community diversity and species richness decreased with increasing soil salinity and pH; in particular, the Shannon–Wiener index decreased with increasing soil salinity and pH in the Cerknica wetland in Slovenia (Gaberscik et al., 2018). Zhang et al. (2019) found that the Simpson dominance index of plant communities decreased with decreasing soil nitrogen content in the Momoge Wetland, China (Zhang et al., 2019a). Although those findings are inconsistent with each other, they all suggest that soil property indices have some relationship with *T. chinensis* community diversity; however, few studies have quantified this relationship from the perspective of comprehensive soil habitat conditions.

It has been hypothesized that soil habitat conditions determine *T. chinensis* community diversity. To explore this, a three-year field investigation of *T. chinensis* community diversity and soil habitat conditions was conducted in the Yellow River Delta, China, from 2020 to 2022. This study aimed to 1) reveal the variation in *T. chinensis* community diversity under various soil habitat conditions and 2) quantify the relationship between *T. chinensis* community diversity and soil habitat conditions. The underlying mechanisms were also investigated.

## 2 Materials and methods

### 2.1 Study area

The Yellow River Delta (E118°10′-119°09′, N37°09′-38°09′, elevation< 15 m) is located at Dongying City, Shandong Province, China. The study area experiences a warm temperate semi-humid continental monsoon climate, with an average annual temperature of 12.6°. The average annual precipitation ranges from 530 to 630 mm, with most of it concentrated during July–August (Zhang et al., 2008). The Yellow River is well-known for its high sediment load, which is deposited along the river banks and delta. This sediment buildup over time has resulted in the creation of an extensive wetland landscape. However, the Delta has also been impacted by seawater intrusion, which has caused the groundwater table to become shallow (< 1.5 m) and the soil to become severely salinized (soil salt content< 6 g kg^-1^) (Yu et al., 2022). In general, the soil salinity decreases, and the groundwater table deepens from the coastline to the inland wetlands, creating a strong soil habitat condition gradient and a plant community successional gradient (Yang et al., 2018). *T. chinensis*, a plant species with high salt tolerance, is a representative species that grows inland from the coast and forms the largest shrub community in the Delta (Hussain et al., 2022). Other plant species such as *Suaeda salsa* (L.) Pall., *Phragmites australis* (Cav.), *Cynodondactylon* (Linn.), *Imperata cylindrica* (Linn. Beauv), and *Cynanchum chinense* also grow in the *T. chinensis* shrubs, increasing the plant species richness and diversity in the *T. chinensis* community.

### 2.2 Field investigation and monitoring

Since soil habitat conditions primarily change with distance from the coastline, three typical and contiguous *T. chinensis* shrubs (tree age of 8–10 years) without the interference of human activities and at three (10, 20, and 40 km) different distances from the coastline were selected for the field study. Before the field study commenced in 2020, two smart sensors (Thermal Dissipation Probe TDP-30, Dynamax Inc., Houston, TX, USA) were installed beneath the shrubs at soil depths of 0–90 cm at the three distances, and soil moisture and EC were automatically recorded at 1 h intervals. The *T. chinensis* shrubs adjacent to the sensors were investigated annually in August 2020, 2021, and 2022 (August is when the shrubs grow most vigorously). First, two random plots (5 × 5 m) were established, the number of *T. chinensis* individuals was determined and their density was calculated in each plot. Five individuals of *T. chinensis* were randomly selected from each plot to measure their average height, canopy diameter, ground diameter, and canopy coverage with a tape measure (Malcolm, 2016). Then, three sub-quadrats (1 × 1 m) were set along the diagonal in each plot, and the plant species, density, and average height of the herbaceous plants were assessed in the sub-quadrats as determining *T. chinensis*. In total, 36 plots were studied during the three-year investigation. Finally, a soil profile of 1.3 m depth was dug in each plot, and the bulk soil cores (100 cm^3^) and soil samples were collected at 10 cm intervals at 0–50 cm soil depth and at 20 cm intervals at 50–130 cm soil depth (Mahaney William and Krishnamani, 2003). The collected soil cores and samples were transported to the laboratory for physical and chemical analyses.

### 2.3 Soil analysis

Bulk soil cores were oven-dried to determine soil bulk density (BD). The soil samples were air-dried and then divided into two sub-samples: one sub-sample was passed through a 2 mm sieve after removing the coarse debris and stones, to determine soil texture, pH, and electrical conductivity (EC); the other sub-samples were passed through a 0.25-mm sieve, to determine soil total nitrogen (Total-N), total phosphorous (Total-P), and total potassium (Total-K). Soil texture was determined using a laser particle size analyzer (Melvin MS3000, Malvern Panalytical, England) after treatment with a dispersant. The pH was determined using a pH meter (PHS-3E, Rex Electric Chemical, Shanghai, China), and the EC was measured using a portable conductivity meter (DDS-307A, Rex Electric Chemical, Shanghai, China) in a 1:5 soil: water solution (Hu et al., 2021). Total-N content was determined using the semi-micro Kjeldahl method (Wikoff and Moraghan, 1985). The Total-P was determined using the sulfuric acid-perchlorate-boiled-molybdenum antimony colorimetric method (Xiao et al., 2012). Finally, the Total-K content was determined using the flame photometer method (Blakemore, 1987).

### 2.4 Plant community diversity

Simpson dominance, Shannon–Wiener, Pielou, and Margalef indices were used to quantify *T. chinensis* community diversity. The Simpson dominance index (C, -) indicates the change in dominant species; the larger the value of C, the greater the dominance of species in the communities. The Shannon–Wiener index (H, -) measures the diversity of species; the higher the H value, the higher the diversity of species in a community. Pielou index (E, -) indicates the relative density of each species in the community; the larger the E value, the more even the distribution of individual species in the community. Margalef index (Ma, -) represents the number of species in a community; the greater the Ma value, the greater the number of species in a community. The four indices are estimated as follows (Eq. 1-5).


(eq. 1) Pi=ni/N

(eq. 2) $$C=\sum^{}Pi^{2}$$

(eq. 3) $$H=-\sum^{}\left(Pi\cdot lnPi\right)$$

(eq. 4) E=H/lnS

(eq. 5) Ma=(S−1)/lnN

where *ni* is the number of individuals of plant species *i*; *N* is the number of individuals of all plant species in a particular quadrat (n); *Pi* is the relative abundance of plant species *i* in a particular quadrat; *S* is the total number of species in the plant community (n).

### 2.5 Data processing

One-way analysis of variance (ANOVA) and the least significant difference (LSD) method were used to compare the differences in soil factors and plant community diversity at the three distances.

Data reduction using principal component analysis (PCA) was performed to quantify the relationship between integrated soil habitat conditions and community diversity at the three distances, following the methods described by Gao et al. (2014). First, Pearson’s correlation analysis was calculated to analyze the correlation between soil factors (all factors were geometric averaged with the soil depth from 0 to 1.3m) and community diversity, and three soil-influencing determinants (sand content, mean soil moisture, and EC) of community diversity were obtained. Second, the KMO and Bartlett tests were performed on soil sand content, soil moisture, and EC, it suggested that the KMO sample fitness test statistic is 0.656, indicating a strong correlation and overlap between variables in this particular case. Additionally, the Bartlett test resulted in a chi-square value of 20.698 and a p-value of 0.00, which is highly significant. Both tests demonstrate that the data is well-suited for principal component analysis (PCA). Third, an integrated soil habitat condition index (SHI) was developed to represent the aggregated soil influencing determinants. Notably, the SHI was a synthetic variable derived from the first principal component of the PCA of the three soil determinants in CANOCO 4.5. It suggests that SHI could explain 64.2% of the variation in the three soil determinants. Third, SHI was used to predict *T. chinensis* community diversity in Origin 2019.

## 3 Results

### 3.1 Soil properties and *T. chinensis* community

Nine soil factors representing soil physics, water, salt, and nutrient properties were significantly different in the *T. chinensis* plots at the three distances from the coastline (*P<*0.05). However, the pH and Total-P content were exceptions to this trend (**Table 1**). Generally, the soil bulk density increased (1.31 ± 0.12–1.55 ± 0.09 g cm^-3^), soil texture became more clayey (5.26 ± 0.00–7.77 ± 0.00%), the soil moisture regime became drier (30.62 ± 5.08–37.32 ± 3.70 cm^3^ cm^-3^), soil salinity was lowered (EC:2.79 ± 0.33 - 5.07 ± 0.31 mS cm^-1^, salt content:3.74 ± 0.91 - 7.35 ± 1.47 g kg^-1^), and soil Total-N became enriched (0.25 ± 0.03 - 0.52 ± 0.21 g kg^-1^) as the distance from the coast increased.

**Table 1:** Selected soil basic physicochemical properties in the investigated *Tamarix chinensis* shrub plots at the three different distances to coastal line.

Distance to coastal line km	Soil physical properties				Soil water and salinity				Soil nutrients		
	Bulk density	Clay	Sand	Silt	Soil moisture	Soil salt content	EC	pH	Total-N	Total-P	Total-K
	g cm^-3^	%	%	%	cm^3^ cm^-3^	g kg^-1^	mS cm^-1^		g kg^-1^	g kg^-1^	g kg^-1^
40	1.55 ± 0.09^a^	7.77 ± 0.00^a^	13.64 ± 0.03^b^	79.35 ± 0.03^a^	30.62 ± 5.08^b^	3.74 ± 0.91^b^	2.79 ± 0.33^a^	7.92 ± 0.43^a^	0.52 ± 0.21^a^	0.53 ± 0.02^a^	16.84 ± 0.33^a^
20	1.34 ± 0.23^b^	5.54 ± 0.00^b^	13.96 ± 0.01^b^	80.39 ± 0.02^a^	33.56 ± 4.58^b^	4.14 ± 1.21^b^	3.08 ± 0.31^a^	7.99 ± 0.50^a^	0.32 ± 0.09^b^	0.54 ± 0.03^a^	14.36 ± 0.89^b^
10	1.31 ± 0.12^b^	5.26 ± 0.00^b^	19.25 ± 0.02^a^	75.16 ± 0.02^b^	37.32 ± 3.70^a^	7.35 ± 1.47^a^	5.07 ± 0.31^b^	7.95 ± 0.28^a^	0.25 ± 0.03^b^	0.54 ± 0.02^a^	17.07 ± 0.56^a^

Different lowercase letters indicate significant differences at *P*<0.05.

*T. chinensis* density, ground diameter, and canopy coverage significantly differed in the investigated plots at the three distances (*P<*0.05); however, the average height and canopy diameter deviated from this trend (**Table 2**). The density (0.55 ± 0.49–1.13 ± 0.77 n m^-2^), ground diameter (41.68 ± 33.03–83.83 ± 48.56 mm), and canopy coverage (31.97 ± 0.18–51.09 ± 0.19%) increased with distance. Different undergrowth herbs grew in the *T. chinensis* shrubs, in which most herb species were found at a distance of 20 km and the least at a distance of 40 km. *Suaeda salsa* (L.) Pall., *Phragmites australis* (Cav.), and *Cynanchum chinense* were detected at all three distances. The highest density of *Suaeda salsa* (L.) Pall. was at the 20 km distance (573.9 n m^-2^) and lowest at the 40 km distance (191.9 n m^-2^).

**Table 2:** Community structure of the *Tamarix chinensis* shrubs in the investigated plots at the three different distances to coastal line.

Distance to coastal line (km)	Shrub (*T. chinensis*)					Grass		
	Density(n m^-2^)	Ground diameter (mm)	Average height (m)	Canopy diameter (m)	Canopy coverage (%)	Species (-)	Density (n m^-2^)	Average height (m)
40	1.13 ± 0.77^a^	83.83 ± 48.56^a^	1.38 ± 0.26^a^	0.92 ± 0.33^a^	51.09 ± 0.19^a^	*Suaeda salsa (L.) Pall.*	465	0.28
						*Phragmites australis (Cav.)*	127.7	0.36
						*Cynodondactylon (Linn.)*	200.3	0.41
						*Cynanchum chinense.*	11	2.01
						*Acorus calamus L.*	132	0.56
						*Setaria viridis (L.)*	14.5	0.42
						*Sonchus wightianus DC.*	2.22	0.27
						*Artemisia annua L.*	0.17	0.86
						*Salsola collina Pall.*	4	0.45
						*Juncus effusus L.*	16	0.47
20	0.55 ± 0.49^b^	57.52 ± 22.51^ab^	1.28 ± 0.29^a^	0.92 ± 0.35^a^	31.97 ± 0.18^b^	*Suaeda salsa (L.) Pall.*	573.9	0.35
						*Phragmites australis (Cav.)*	25.36	0.53
						*Cynodondactylon(Linn.)*	144.5	0.47
						*Cynanchum chinense.*	9.67	0.52
						*Acorus calamus L.*	27	0.87
						*Setaria viridis (L.)*	22.5	0.5
						*Sonchus wightianus DC.*	16.8	0.32
						*Artemisia annua L.*	42	0.71
						*Imperata cylindrica (Linn.Beauv.)*	60	0.66
						*Gramineae Aeluropus Angiospermae*	624	0.42
						*Heteropappus hispidus (Thunb.)*	2	0.4
						*Artemisia capillaries*.	1	0.59
						*Tripolium vulgare Nees*	4	0.76
						*Paspalum thunbergii Kunth.*	9.5	0.39
10	0.64 ± 0.53^ab^	41.68 ± 33.03^b^	1.19 ± 0.49^a^	1.00 ± 0.51^a^	33.05 ± 0.14^b^	*Suaeda salsa (L.) Pall.*	191.9	0.34
						*Phragmites australis (Cav.)*	73.4	0.7
						*Cynanchum chinense.*	32	0.77
						*Acorus calamus L.*	256	0.6
						*Sonchus wightianus DC.*	16	0.27
						*ApocynumvenetumL.*	16	1.08
						*Limonium bicolor (Bag.)*	14.5	0.14

### 3.2 *Tamarix chinensis* community diversity

Simpson dominance, Margalef, and Pielou indices showed significant differences in the *T. chinensis* plots at the three distances (*P<*0.05), but the Shannon–Wiener index did not (**Table 3**). Simpson dominance index (0.403 ± 0.03–0.641 ± 0.02) significantly and sequentially increased at 40, 20, and 10 km from the coastline (*P<*0.05). Pielou index was the lowest at the 20 km distance (0.329 ± 0.08) and the highest at the 10 km distance (0.657 ± 0.05). Margalef index was the highest at a distance of 20 km (0.446 ± 0.06) and lowest at 10 km distance (0.351 ± 0.11).

**Table 3:** Species diversity in the investigated *Tamarix chinensis* plots at the three different distances to coastal line.

Distance to coastal line (km)	Simpson dominance index	Shannon–Wiener index	Pielou index	Margalef index
40	0.403 ± 0.03^c^	0.671 ± 0.07^a^	0.543 ± 0.06^ab^	0.376 ± 0.06^a^
20	0.485 ± 0.03^b^	0.617 ± 0.06^a^	0.329 ± 0.08^b^	0.446 ± 0.06^b^
10	0.641 ± 0.02^a^	0.714 ± 0.11^a^	0.657 ± 0.05^a^	0.351 ± 0.11^a^

**Figure 1:**
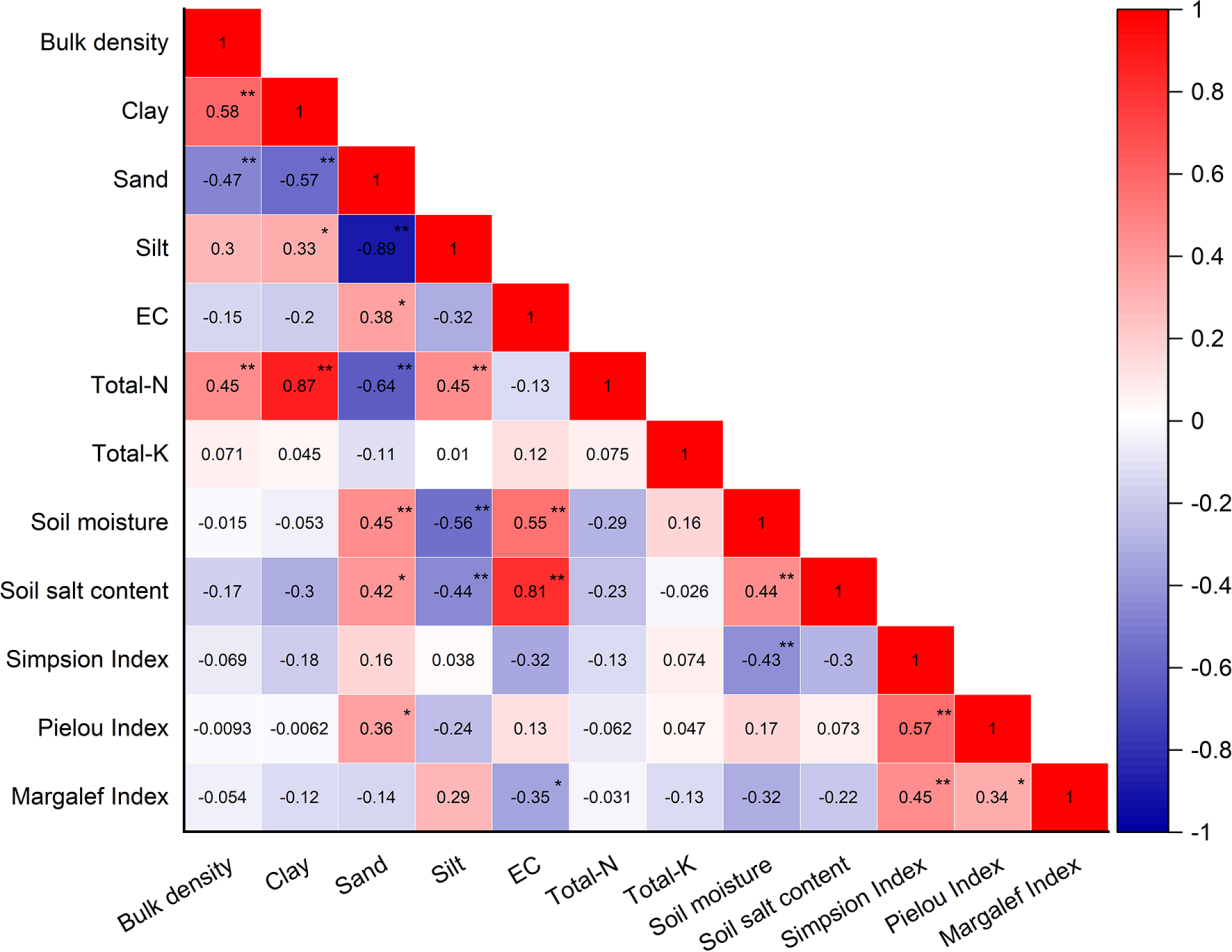
Correlation analysis of selected basic soil properties, soil moisture, soil salt content, and species diversity in *Tamarix chinensis* shrub plots. * indicate significant differences at P<0.05, and ** indicate significant differences at P<0.01.

**Figure 2:**
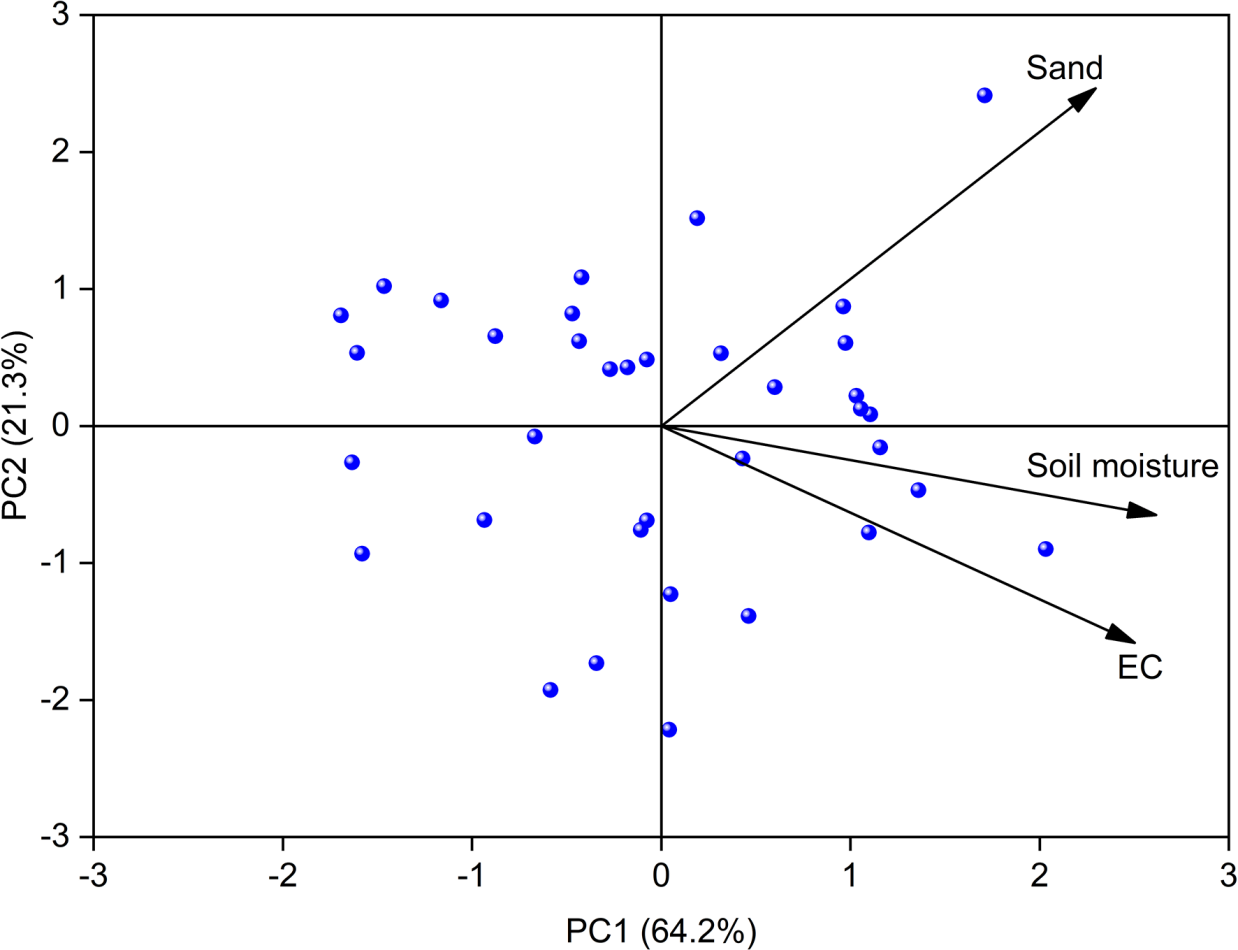
Principal component analysis (PCA) of selected basic soil properties, soil moisture, soil salt content, and species diversity in *Tamarix chinensis* shrub plots.

**Figure 1** shows that the *T. chinensis* community diversity was significantly correlated with sand content, mean soil moisture, and EC in the above-mentioned nine soil factors (*P<*0.05). Simpson dominance index was significantly and negatively correlated with mean soil moisture (r = -0.43, *P<*0.05). The Pielou index had a significant and positive correlation with sand content (r = 0.36, *P<*0.05), whereas the Margalef index was significantly and negatively correlated with EC (r = -0.35, *P<*0.05). The principal component analysis (PCA) was implemented to develop an integrated soil habitat index in the *T. chinensis* shrubs (**Figure 2**). The SHI explained 64.2% of the variation in the soil habitat conditions.

### 3.3 Soil habitat indices regulating *T. chinensis* community diversity

**Figure 3:**
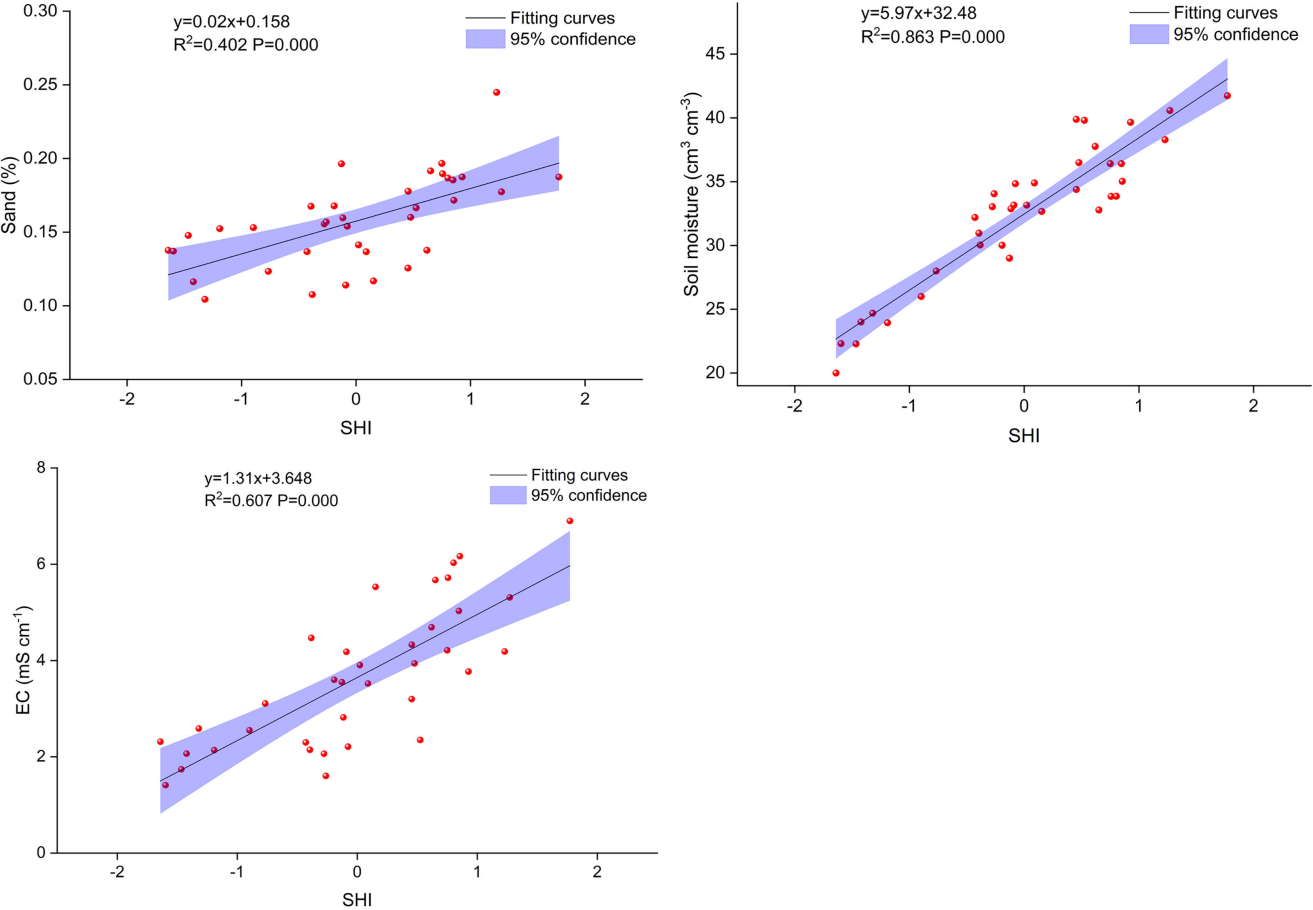
Relationship between soil properties and soil habitat index (SHI) in *Tamarix chinensis* shrubs.

**Figure 4:**
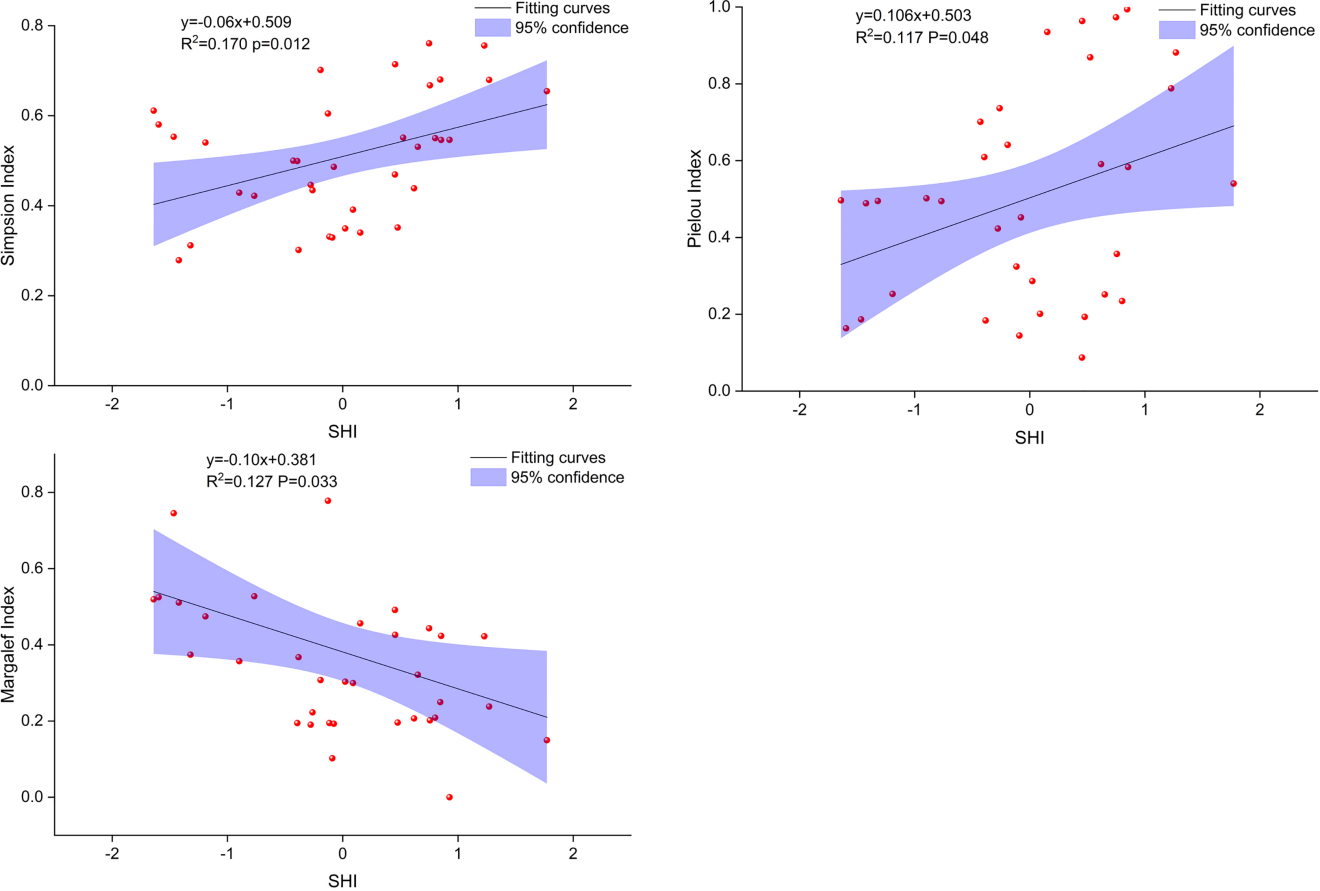
Predicting species diversity in the *Tamarix chinensis* shrubs plots based on the integrated soil habitat index (SHI).

**Figure 5:**
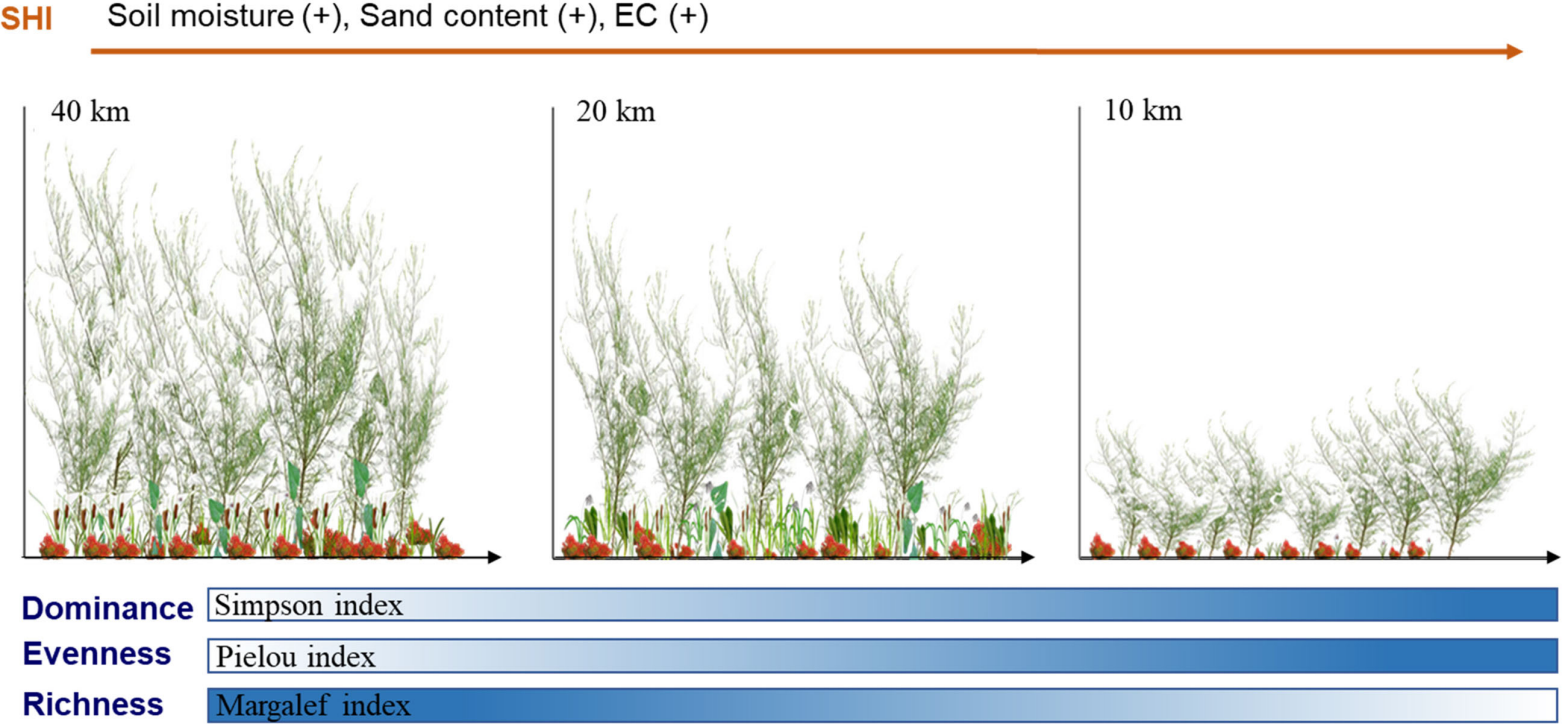
Soil habitat indices regulate the development of species diversity in *Tamarix chinensis* shrubs in coastal saline regions.

Soil habitat indices were significantly correlated with sand content, mean soil moisture, and EC in *T. chinensis* shrubs (**Figure 3**). The SHI was positively correlated with sand content, mean soil moisture, and EC at *p* = 0.00 level (R^2 =^ 0.40–0.86) and increased with the increase in the three layers. Moreover, the SHI could predict *T. chinensis* community diversity at different distances from the coastline (**Figure 4**), with the determination coefficient (R^2^) ranging from 0.12 to 0.17 and the *p*-value ranging from 0.01 to 0.05. The SHI indices showed that *T. chinensis* community diversity changed substantially. As **Figure 5** illustrates, with decreasing distance from the coastline, the SHI increased. On the same gradient, Simpson dominance and Pielou indices increased gradually, whereas Margalef indices decreased.

## 4 Discussion

### 4.1 Variations in soil habitat conditions

Field monitoring and investigation suggested a substantial variation in soil habitat conditions at the three distances from the coastline (*P<*0.05) (**Table 1**). Sand content was significantly lower, clay content was significantly higher, and silt content was higher at distances of 40 and 20 km than at distances of 10 km. One reason for the trend is that the coastal soil is mainly affected by tidal action, and fine soil particles are prone to erosion; another is that thicker vegetation benefited soil conservation in inland areas (Nguyen et al., 2012; Yu et al., 2014; Xia et al., 2020). The coarser texture of the soil resulted in a decrease in soil bulk density, particularly as the distance from the coastline decreased. Furthermore, soil moisture levels increased progressively as the distance from the coast decreased, primarily due to the intensification of tidal intrusion, which raised the water table (Rossetti and Scotton, 2017). As salt-enriched shallow groundwater can rapidly rise to the upper soil layers under the action of soil capillary force and evapotranspiration, soil salt content increases with decreasing distance from the coastline (Chi et al., 2020; Fu et al., 2020). At a distance of 40 km, soil Total-N (0.52 ± 0.21 g kg^-1^) was similar to that found under the *T. chinensis* shrub in the lower reaches of Heihe River (0.47–0.53 g kg^-1^) but lower at the 20 and 10 km distances (0.32 ± 0.09, 0.25 ± 0.03 g kg^-1^) (Ding et al., 2018; Berendse et al., 2021). A possible explanation for this trend is that high soil salinity reduced rates of net N mineralization fluxes and turnover, reducing soil nitrogen content (Yu et al., 2016; Ding et al., 2021). However, because soil P and K mainly come from river sediment from the upstream Yellow River, Total-P and Total-K content remained relatively stable on a long-term scale (Xie et al., 2020; Wei et al., 2021), and soil Total-P and Total-K showed no significant differences at the three distances.

### 4.2 *T chinensis* community diversity

The growth form of *T. chinensis* significantly differed at the three distances from the coastline (*P<*0.05) (**Table 2**), suggesting strong effects of soil habitat conditions on the plant community. Plant density and canopy coverage were the highest at 40 km and lowest at 20 km, respectively. In areas with high salinity, populations of *T. chinensis* exhibit improved survival and competitiveness by forming patches with a high degree of aggregation, which enabled them to occupy favorable spaces. This phenomenon is demonstrated by the Allee effect (Jonathan et al., 2017), and it is likely the reason why the density of *T. chinensis* was higher at a distance of 10 km from the coastline, as opposed to 20 km. Cao et al. (2020) reported that *T. chinensis* density and canopy coverage were significantly and negatively correlated with the EC (9.65–25.38 mS cm^-1^). Our results differed, which can probably be attributed to the lower soil EC (2.79 ± 0.33–5.07 ± 0.31 mS cm^-1^). Alternatively, other factors (e.g., soil pH and nutrient status) apart from soil salinity play a role in determining plant density and canopy coverage (Calvo-Polanco et al., 2017). The ground diameter increased with increasing distance from the coastline, in line with previous findings (Cao et al., 2020; Yousaf et al., 2021). The average height decreased with shorter distances to the coastline but did not differ significantly at the three distances. The degree of tolerance of plants to salt stress is mainly reflected by their height (Liu et al., 2014). Although *T. chinensis* is a salt-producing halophyte that secretes salt *in vitro* to avoid excessive salt accumulation and thereby maintain normal physiological activities (Zhang et al., 2019b), the high salt content in its tissues still has an inhibitory effect on height.

Various herbaceous plants grew under the *T. chinensis* canopy (**Table 2**). *Suaeda salsa* (L.) Pall and *Phragmites australis* (Cav.) were found at all three distances, and their density was greater than that of other herbaceous species because of their strong salt tolerance and adaptability (maximum salt tolerance: 25 g kg^-1^, 10 g kg^-1^, respectively) (Flowers and Colmer, 2015). With low salt tolerance, *Salsola collina* Pall and *Juncus effusus* L. only grew at a distance of 40 km. Closer to the coastline, these plants became degraded due to the increasing soil salinity and soil P contents, affecting the *T. chinensis* community diversity (Xia et al., 2016b; Jia et al., 2018). Notably, the Simpson dominance index (indicating changes in the dominant species), Pielou, and Margalef indices showed significant differences between the three distances (**Table 3**). The Simpson dominance index increased closer to the coast because the high soil salinity exerted pressure on plant metabolic processes; therefore, only plants (e.g., *Suaeda salsa* (L.) Pall. and *Phragmites australis*) with high salt tolerance survived and grew among the *T. chinensis* shrubs (Xia et al., 2016a). The Pielou index quantifies the evenness of distribution of individual species in a community. As illustrated in **Figure 1**, the Pielou index increases with increasing sand content (**Figure 1**). Sandy soils have lower nutrient and water-holding capacities than clayey soils (Frédéric et al., 2020), which triggers fierce interspecies competition due to nutrient and water scarcity (Xue et al., 2016; Li et al., 2018). Only plants with a high tolerance to scarcity can survive, and the evenness of plant distribution in the community is high; for instance, the Pielou index was highest at a distance of 10 km. The Margalef Index determines the number of species in a community. The Margalef index was significantly and negatively correlated with the EC, which is consistent with previous findings (Zhang et al., 2018). As a result, the Margalef index was found to be the lowest at a distance of 10 km from the coastline. In addition, the Margalef index was observed to be higher at a distance of 20 km compared to 40 km. It is possible that other soil properties influenced the Margalef index at the 20 km distance, as evidenced by the fact that the EC only accounted for 35% of the variation observed in the Margalef index (**Figure 1**). This highlights the significance of integrated soil habitat conditions in determining the diversity of the *T. chinensis* community. The Shannon–Wiener index quantifies species diversity. In our study, plant diversity was low because salt secretion by *T. chinensis* leads to the accumulation of soil salt under the *T. chinensis* canopy, inhibiting the growth of herbaceous undergrowth (Tao et al., 2022a). Therefore, the Shannon–Wiener index showed no significant differences among the three distances.

### 4.3 Soil habitat condition predicting *T. chinensis* community diversity

The estimated soil habitat index (SHI) quantified 64.2% of the variables of sand, soil moisture, and EC (**Figure 2**) and was thus an appropriate quantification of the soil habitat condition of the *T. chinensis* community. Existing literature largely reports that soil texture, soil moisture, and EC are the main determinants of *T. chinensis* community diversity in the Yellow River Delta (Xia et al., 2016a; Li and Liu, 2020; Yang et al., 2021; Chen et al., 2022). The SHI eliminated the multicollinearity of the determinants and improved the quantification of the soil habitat conditions. We found that SHI was significantly and positively correlated with sand content, mean soil moisture, and EC. This means that the coarser the soil texture, the wetter the soil moisture regime, and the higher the soil salinity, the greater the SHI value. The SHI accurately predicted *T. chinensis* community diversity (R^2 =^ 0.12–0.17, *p* = 0.01–0.05) (**Figure 3**). Wang et al. (2019) predicted the four plant diversity indices under different degradation degrees (R^2 =^ 0.18 to 0.58, *p* = 0.00–0.09), and Wu et al. (2018) predicted the Margalef index of different plant communities by linear fitting (R^2 =^ 0.25 to 0.82, *p* = 0.00) (Wu et al., 2018; Wang et al., 2019). Although these models performed well, they were only able to predict individual soil factors affecting plant community diversity.

Numerous studies have found that a variety of soil determinants change plant community diversity and have used the results to develop a series of predicting models (Wubs and Bezemer, 2018; Yi et al., 2020). Our study integrated soil determinants into the SHI to quantify soil habitat conditions, which eliminated the determinants’ multicollinearity and improved the accuracy of the prediction model. The prediction models showed that when the SHI increased with decreasing distance to the coastline, the Simpson dominance and Pielou indices increased gradually, while the Margalef indices decreased (**Figure 5**). These findings remind us that the effect of soil determinants on the variation in plant community diversity is not independent and that this effect should be considered comprehensively, as manifested in the presentation of SHI in the Yellow River Delta. Creating an optimal soil health index (SHI) will promote the development of the *T. chinensis* community and enhance its ecological functions. However, given that plants and soil are commonly co-evolutionary, further studies are needed to elucidate the interactions between *T. chinensis* communities and SHI, the understanding of which is crucial for the restoration and conservation of *T. chinensis* communities in the Yellow River Delta.

## 5 Conclusions

Our three-year field investigation showed that the heterogeneity of soil habitat factors significantly influenced the growth of *T. chinensis* and its community diversity in the Yellow River Delta. *T. chinensis* density, ground diameter, and canopy coverage significantly increased with increasing distance, whereas most plant species were detected at a distance of 20 km and the least at 10 km. The Simpson dominance, Margalef, and Pielou indices significantly differed among the three distances, but the Shannon–Wiener index did not. The SHI was constructed to quantify soil habitat conditions in the *T. chinensis* communities using principal component analysis (PCA). The estimated SHI quantified 64.2% of the soil habitat conditions. The SHI was significantly higher at 10 km than at 40 and 20 km. The coarser the soil texture and wetter the soil moisture regime, and the higher the soil salinity, the greater the SHI value. The SHI linearly predicted *T. chinensis* community diversity at the three distances, highlighting the importance of the integrated effects of soil texture-water-salinity condition on plant community diversity. These results can be used in planning the restoration and protection of *T. chinensis* communities in terms of soil habitat conditions in the Yellow River Delta. However, because plants and soils are coevolutionary, further studies are needed to elucidate the interactions between plant communities and soil habitat indices, which are crucial to ensure the success of restoration and conservation projects for plant communities in the Yellow River Delta.

## Data availability statement

The original contributions presented in the study are included in the article/supplementary material. Further inquiries can be directed to the corresponding author.

## Author contributions

YW is responsible for the paper’s ideas and framework as well as the revision of the paper. QY was responsible for collecting and analyzing the data and drafting the paper. LS, QH and JQ proposed the research idea and designed the research scheme. YS was responsible for the determination of experimental data. YZ participated in the revision of the paper. All authors contributed to the article and approved the submitted version.
